# Classification of collagen remodeling in asthma using second-harmonic generation imaging, supervised machine learning and texture-based analysis

**DOI:** 10.3389/fbinf.2025.1539936

**Published:** 2025-04-17

**Authors:** Natasha N. Kunchur, Joshua J. A. Poole, Jesse Levine, Tillie-Louise Hackett, Rebecca Thornhill, Leila B. Mostaço-Guidolin

**Affiliations:** ^1^ Department of Systems and Computer Engineering at Carleton University, Ottawa, ON, Canada; ^2^ Anesthesiology, Pharmacology and Therapeutics Department at the University of British Columbia, Medical Sciences, Vancouver, BC, Canada; ^3^ Department of Radiology, Radiation Oncology, and Medical Physics at the University of Ottawa, Ottawa, ON, Canada; ^4^ Department of Medical Imaging at the Ottawa Hospital, Ottawa, ON, Canada

**Keywords:** airway remodeling, asthma, collagen, machine learning, second harmonic generation, texture analysis

## Abstract

Airway remodeling is present in all stages of asthma severity and has been linked to reduced lung function, airway hyperresponsiveness and increased deposition of fibrillar collagens. Traditional histological staining methods used to visualize the fibrotic response are poorly suited to capture the morphological traits of extracellular matrix (ECM) proteins in their native state, hindering our understanding of disease pathology. Conversely, second harmonic generation (SHG), provides label-free, high-resolution visualization of fibrillar collagen; a primary ECM protein contributing to the loss of asthmatic lung elasticity. From a cohort of 13 human lung donors, SHG-imaged collagen belonging to non-asthmatic (control) and asthmatic donors was evaluated through a custom textural classification pipeline. Integrated with supervised machine learning, the pipeline enables the precise quantification and characterization of collagen, delineating amongst control and remodeled airways. Collagen distribution is quantified and characterized using 80 textural features belonging to the Gray Level Cooccurrence Matrix (GLCM), Gray Level Size Zone Matrix (GLSZM), Gray Level Run Length Matrix (GLRLM), Gray Level Dependence Matrix (GLDM) and Neighboring Gray Tone Difference Matrix (NGTDM). To denote an accurate subset of features reflective of fibrillar collagen formation; filter, wrapper, embedded and novel statistical methods were applied as feature refinement. Textural feature subsets of high predictor importance trained a support vector machine model, achieving an AUC-ROC of 94% ± 0.0001 in the classification of remodeled airway collagen vs. control lung tissue. Combined with detailed texture analysis and supervised ML, we demonstrate that morphological variation amongst remodeled SHG-imaged collagen in lung tissue can be successfully characterized.

## 1 Introduction

ASTHMA is characterized by chronic airway inflammation (the target of all current asthma therapeutics) and airway remodeling, which involves the epithelium, basement membrane, lamina propria, smooth muscle and vascular structures (Holgate, 2008). Airway remodeling was first denoted to be present in fatal asthmatics by Hubert and Kossler in 1922 and since then, has been documented in all stages of asthma severity (Huber, 1922). Airway remodeling has also been linked to reduced lung function, airway hyperresponsiveness and greater use of asthma medications (Bergeron et al., 2010; Boulet et al., 1997; Elias et al., 1999; James et al., 2002; Zeiger et al., 1999). Increased deposition of fibrillar collagens-I and III has been shown within the extracellular matrix (ECM) remodeled airways of asthmatic patients (Dunnill, 1960).

There is a natural abundance of fibrillar collagen within the ECM of the lung to retain tensile strength and support suitable architecture. However, alterations in the concentration and distribution of collagen fibres can lead to loss of anatomical structure, compromised cell function, and tissue fibrosis (Buehler, 2006; Engler et al., 2009; Hoshino et al., 2010; Liu L. et al., 2021).

To understand the changes in ECM at a microcellular level, tissue analysis is conducted ex-vivo. Historically, histological staining has been the predominant approach for studying the complexity of airway remodeling and for visualizing alterations in cell behaviour and tissue structure (Dunnill, 1960; Mostaço-Guidolin et al., 2021). The abundant proliferation of collagen in asthmatic airway remodeling was first visualized using Verhoeff-van Gieson and Masson trichrome staining (Carroll et al., 2000; Dunnill, 1960; Hogg, 1997). While histological staining remains the gold standard for assessing tissue alterations, the process of staining and sample fixation often alters the tissue structure through protein denaturation and cross-linking (Alturkistani et al., 2015; Javaeed et al., 2021). Furthermore, the representation of the collagen fibers are mixed with other extracellular components, making it difficult to resolve exact collagen proteins within a sample when applying H&E staining (Keikhosravi et al., 2020). These traditional staining methods lack specificity for detailing ECM proteins’ biochemical and structural information, particularly the fibrillar collagen deposited during airway remodeling.

A non-centrosymmetric structure in crystallography refers to a crystal structure that lacks a center of symmetry. In such structures, there is no point within the crystal through which every part of the structure has an identical part located symmetrically opposite. This lack of inversion symmetry often leads to unique physical properties, such as piezoelectricity, second-harmonic generation, and other nonlinear optical effects.

Fibrillar collagen lacks a centre of symmetry, demonstrating a non-centrosymmetric property. This lack of inversion symmetry leads to its unique physical properties, making it an ideal candidate for nonlinear optical effects such as second harmonic generation (SHG) imaging (Bancelin et al., 2014; Chen et al., 2012; Cox et al., 2003; Han et al., 2005; Poole Mostaço-Guidolin et al., 2021). Since the first demonstration of the SHG application and its capabilities in imaging biomolecules, it has been widely used to monitor fibrillar collagen and its role in remodeling and wound repair (Deka et al., 2012; Israel et al., 2017; Sun et al., 2008; Tilbury et al., 2014). SHG allows for label-free tissue structure assessment without requiring exogenous labelling and/or extensive sample preparation. Visualizing fibrillar collagen through SHG imaging has provided insight into the morphological alterations in asthmatic lung tissue (Mostaço-Guidolin et al., 2019).

With the promise of non-invasive imaging, SHG is capable of visualizing freshly obtained tissues in their natural physiological state. With this, the structural information of the samples can be accessed immediately. Additionally, when combining fixation methods such as paraffin embedding with SHG microscopy, one can achieve prolonged storage of tissue, increasing SHG microscopy versatility to image fresh or preserved tissues. Fixation allows for maintaining and highlighting the integrity of tissue morphology. Furthermore, paraffin-embedding supports the serial sectioning of tissues, allowing for detailed cross-sectional analysis of tissues, inclusive of airways. This routinely adopted method can guide digital reconstruction of 3D data and visualize the intricate anatomy of the lungs (the bronchioles, alveoli and alveolar capillary network) (Baschong et al., 2001; Grothausmann et al., 2017; Monaghan et al., 2016). Furthermore, paraffin-fixed tissues allow for successful reinvestigation of archived human tissue. Authors Bredfeldt et al., follow a similar approach, where collagenous fibres of cancerous breast-tissue are evaluated using SHG upon having been paraffin-embedded and H&E stained (Bredfeldt et al., 2014). Different groups have explored and tested the feasibility of using paraffin-embedded tissues for both stained-based microscopy and label-free imaging (Martin et al., 2013; Sabo et al., 2022; Vijayaraghavan et al., 2014). Findings support the use of paraffin-guided fixation agents, countering the common perception that these tissue additives introduce harmful autofluorescence when imaging. Sabo et al., successfully retrieve specialized high-content data from paraffinized tissue sections when measuring collagen distribution in interstitial fibrosis with SHG imaging (Sabo et al., 2022).

However, to fully capitalize on the intricate level of detail offered by SHG imaging, effective pattern recognition tools are required to appreciate the specific features associated with tissue remodeling. Gray-level textures represent an advanced statistical approach for quantifying the spatial distribution of gray-level pixel intensities within a localized area (Liu Y. et al., 2021; Song et al., 2022). With the organization of pixel intensities into a tabular format, various features detailing the structural properties of the SHG-imaged tissue can be derived through the quantitative distribution of pixels. We explore the application of five primary texture groups: Gray Level Cooccurrence Matrix (GLCM), Gray Level Size Zone Matrix (GLSZM), Gray Level Run Length Matrix (GLRLM), Gray Level Dependence Matrix (GLDM), and Neighboring Gray Tone Difference Matrix (NGTDM) (Amadasun and king, 1989; Galloway, 1975; Haralick et al., 1973; Sun and Wee, 1983; Thibault et al., 2014). Through the examination of pixel arrangement, gray-level textures can help infer the underlying organization and structure of collagen fibrils (Cicchi et al., 2013; Hu et al., 2012; Wu et al., 2016). The extracted textural features are used to train a support vector machine capable of differentiating between control and remodeled asthmatic airways. In automating the classification of SHG images based on remodeling status, we present a quantitative approach for monitoring changes using gray-level textures. We demonstrate SVM’s robust capabilities as a powerful classification tool adept at discerning morphology types within collagen fibrils, while successfully accounting for minute differences easily missed by the human eye. This method offers scalability, allowing for its extension across various contexts and pathologies.

With the interactions between the microenvironment and its cellular components slowly becoming understood as a driving force in several chronic diseases, our work presents a methodology capable of visualizing these imposed structural changes during the onset of asthma. Our developed pipeline proposes a proof-of-concept that explores the non-invasive characterization of tissue remodeling and paves the way to assess detail regarding ECM structure alterations which are not possible to track using common staining strategies. An increased understanding of the ECM and its contribution in chronic lung diseases can support the testing and development of novel targeted therapies. While leveraging SHG microscopy and robust image analysis methods, we propose a novel image-based, reproducible quantitative methodology pipeline, capable of exploring diseased microenvironments and tracking changes associated to tissue structure alterations due to diseases and/or therapeutic interventions.

## 2 Materials and methods

### 2.1 Human lung tissue preparation and dataset

A total of 12 lungs deemed unsuitable for transplantation were obtained with informed consent from next-of-kin of donors with asthma (n = 6) and donor controls (n = 6) through the International Institute for the Advancement of Medicine (IIAM, Edison, NJ; www.iiam.org) and biobanked within the James Hogg Lung Registry (Ethics protocol number H00-50110). This study was approved by the Providence Healthcare Research Ethics Board (H13-02173). As previously described, the lungs were air-inflated to 10 cmH2O, and frozen over liquid nitrogen vapour. Lungs were then cut into 2 cm thick slices (transaxial plane) (Hogg et al., 2004; Mostaço-Guidolin et al., 2019) and each slice was uniformly randomly sampled by using a line grid (225 mm^2^), superimposed onto the photographs of each lung slice. Sampled cylindrical tissue cores (15 × 20 mm) were formalin-fixed and paraffin-embedded. Sequential 5 μm tissue sections containing airways were used for SHG imaging, with sectioned counterparts stained with Verhoeff-van Gieson stain and digitally scanned (ScanScope XT slide scanner; Aperio Technologies) for reference. Through sequential slicing, the orientation of the tissue sessions remained consistent throughout imaging. Of the total dataset of 549 images; 296 are representative of asthmatic donors and 253 representative of control. The exact image collection amongst control and asthma is as follows: control (31, 37, 37,43,47, 58) and asthma (43, 55, 38, 39, 56, 65).

### 2.2 Second harmonic generation (SHG) imaging

Each unstained tissue section was imaged using a multimodal Nonlinear Optical microscope (NLOM), housed at the National Research Council in Winnipeg, Canada. The custom-built system allowed for the acquisition of correlative two-photon excitation fluorescence (TPEF), Coherent Anti-Stokes Raman Scattering (CARS) and SHG imaging, as previously described (Ko et al., 2009; Ko et al., 2010). A Ti:Sapphire oscillator (Tsunami, Spectra-Physics) operating at 800 nm with a 100 fs pulse duration and 20 nm bandwidth was used. The backward SHG signal was collected and transmitted to a non-descanned PMT detector (H9656 series, Hamamatsu, Bridgewater, NJ, USA) mounted on the microscope assembly (400 ± 30 nm), using a 20x, 0.75NA infinity corrected air objective lens (Olympus Canada, Markham, ON, Canada). The system generated images of approximately 450 × 450 μm with a translational resolution of 0.2 μm. A motor controller (MP-285, Sutter Instrument, Novato, CA, USA) provided the motorized translational movement of the stage (Sutter Instrument, Novato, CA, USA). ScanImage (ver.3.5) software was used for image acquisition and laser scanning control (Pologruto et al., 2003). The typical pixel dwell time for an average of four scans for a single frame collection was 21μs.

### 2.3 SHG image classification pipeline overview

**FIGURE 1:**
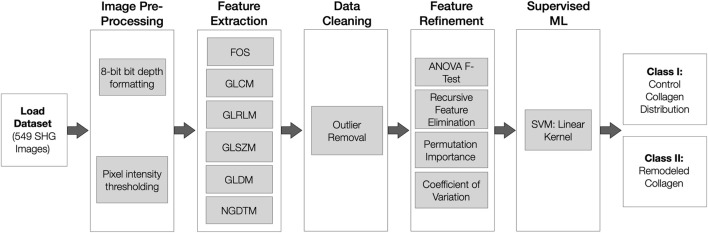
Overview of SHG Image Classification Pipeline. Inclusive of five sequential stages: image dataset pre-processing, textural features extraction, data cleaning, feature refinement and morphology classification using a supervised SVM model. SHG: Second Harmonic Generation. SVM: Support Vector Machine. FOS: First Order Statistics. GLCM: Gray-level cooccurrence matrix. GLSZM: Gray Level Size Zone Matrix. GLRLM: Gray Level Run Length Matrix. GLDM: Gray Level Dependence Matrix. NGTDM: Neighbouring Gray Tone Difference Matrix.

A total of 549 labelled SHG images of airways were obtained and used in this study. The differences in signal distribution patterns associated with collagen fibre deposition were characterized using the image classification pipeline shown in Figure 1. The developed pipeline is comprised of five chronological steps: image preprocessing, feature extraction, data cleaning, feature refinement and classification. To support adequate textural analysis, image preprocessing first ensures the SHG images are of 8-bit bit-depth and TIFF format, preserving the quality of the graphics. To represent a standardized intensity of SHG fibrillar collagen signal amongst all included images, the dataset had to meet a minimum pixel intensity threshold. For each of the images, 5 gray-level texture matrices are obtained in parallel, extracting relevant features capable of capturing the unique pixel arrangement and characteristics of collagen distribution. The extracted features are sequentially cleaned, replacing outlier data with median values. A total of 80 features were extracted per image and refined using i) ANOVA F-test, ii) recursive feature elimination, iii) permutation importance and iv) coefficient of variation strategies. Lastly, the refined features were supplied into two supervised machine learning models in parallel; testing which framework achieved the highest classification accuracy when discriminating amongst control and remodelled airway tissue.

### 2.4 Image preprocessing

In this study, SHG images originally exported as 16-bit TIFF files were converted to 8-bit in ImageJ (Schneider et al., 2012), for texture analysis, reducing both computational demands and memory usage while still preserving the core textural information (Bankfield, 2024). Although this down conversion restricts the dynamic range, it was deemed acceptable given our focus on relative texture features rather than absolute intensities. A predefined intensity threshold (I < 5) was then applied to exclude low-signal images that lacked sufficient grey-level information for reliable textural analysis. To address outlier values, we replaced anomalous intensities with the median value of the dataset; although alternative methods exist, median replacement was selected for its simplicity and reproducibility when processing large image sets.

### 2.5 Feature extraction and data cleaning

To evaluate the textural features of collagen distribution, a total of 80 gray-level texture features were extracted from each image, including four first-order statistic (FOS) features, 24 Gray-level cooccurrence matrix (GLCM) features, 16 Gray Level Size Zone Matrix (GLSZM) features, 16 Gray Level Run Length Matrix (GLRLM) features, 15 Gray Level Dependence Matrix (GLDM) features, and 5 Neighboring Gray Tone Difference Matrix (NGTDM) features. The matrix operations and the respective features extracted for each family of texture features are defined in Supplementary Table S1. Gray-level texture analysis was performed computationally in two steps: i) base construction of each of the gray-level texture matrices, and ii) calculation of features from each of the matrices. For the GLCM and GLRLM, all features extracted were averaged from four directional matrices at 0°, 45°, 90° and 135°. To address outlier cases, the interquartile range method (IQR) was applied due to its simplicity and low susceptibility to noise (Barbato et al., 2011). While effective for distributions with high skew, it lacks the adaptability to individual cases that other outlier rejection methods might possess. Given the inability to assume that each feature’s distribution is free of high skew, the IQR method emerged as a suitable choice for outlier rejection. Rather than outright removal from the dataset, rejected outliers were substituted with the median value. The median is a robust measure of central tendency and does not shift significantly with outliers such as the mean. Bias is minimal when using median values as the replacement value is independent of skew, preserving the original data structure. As our data is non-normally distributed, using the median ensures that the imputed values are representative of the data. Furthermore, the median value accommodates both small and large datasets (Maronna et al., 2006). This approach is aimed at preserving individual data points and not further limit the size of our dataset. With a comprehensive initial list of 80 texture features extracted from the SHG dataset, we next focus on dimensionality reduction and feature refinement to avoid overfitting and ensure our models capture the most predictive information, as detailed in Section F. This preserves data quality, integrity and further support the reproducibility of our methodology. All methods applied to feature extraction and data cleaning were performed using custom “in-house” Python scripts.

### 2.6 Feature refinement

With 80 features, dimensionality reduction and feature refinement are required to avoid overfitting and to allow adequate generalizability of our collagen classification models. To ensure the high predictive power of the supplied features, four feature refinement methods were investigated: ANOVA F-test (filter method), recursive feature elimination (wrapper method), permutation method (embedded method) and the novel coefficient of variation (statistical) method. The ANOVA F-Test identifies features with the highest ratio of variance between different groups and within the same groups. Distributions that exhibit minimal overlap amongst features are selected, to provide the model with distinctive features that can facilitate easier discrimination between two groups of data (Dissanayake Md Johar, 2021; Remeseiro and Bolon-canedo, 2019). The top ten features ranked using ANOVA F-test scores were selected. Recursive Feature Elimination (RFE) deploys all features initially into a decision tree model, progressively removing one feature at a time while re-fitting the defined decision tree model (Guyon et al., 2002). The iterative process continues until a drop in model accuracy is observed, indicating that the optimal number of features has been reached. Alternatively, permutation importance (PI) determines the optimal feature subset by establishing a baseline score through the training of a random forest classifier. During training, the model is continuously fitted to accommodate a feature which has been “permuted/rearranged”. The random forest classifier is employed to make predictions using this permuted validation data and re-evaluated (Altmann et al., 2010). The PI score for a feature is computed as the difference between the baseline score and the score achieved when the model predicts using the permuted feature. This permutation process is repeated for each feature over 100 iterations to determine which features, on average, cause the most significant changes in accuracy (Altmann et al., 2010). Lastly, a novel Coefficient of Variation (CV) method was applied to capture interclass feature variation. The CV reflects the standard deviation from the mean, where a higher CV signifies a greater dispersion of the mean. As demonstrated in a previous application (Kunchur et al., 2022), a CV threshold of 8% was applied. The detailed methodology of the CV method can be found elsewhere. The subsets of features extracted from each refinement technique were independently used to train a support vector machine model. ANOVA F-test, PI and RFE feature refinement were implemented using Scikit Learn packages in Python (scikit-learn, developers BSD L, 2007a; scikit-learn, developers BSD L, 2007b; scikit-learn developers, BSD License, 2007; scikit-learn, developers BSD L, 2007c)

### 2.7 Supervised machine learning

We trained support vector machine models to delineate SHG images obtained from non-asthmatic controls from those extracted from remodeled (asthmatic) airways. Through the optimization of a hyperplane providing maximal class separation, SVMs have proven to be powerful binary classification models. The default parameters of the sklearn.svm model with a linear kernel were used (scikit-learn, developers BSD L, 2007c). Both linear and RBF kernels were compared, however, linear presented stronger classification metrics. With a balanced dataset, the class weight function was set to a default parameter of one. Linear discriminant analysis was used to further reduce the dimensionality of the feature set for each of the feature refinement methods prior to SVM training. Feature refinement methods and LDA were used in unison to reduce redundant/overlapping features amongst the five textural matrices employed. Feature refinement methods were leveraged with the intention to remove similar features; with LDA then identifying the best discriminative space. When used in combination, higher classification metrics (the ones presented) were observed. The accuracy of each model was assessed through Repeated Stratified K-Fold validation, employing ten splits and conducting three runs per split. Accuracy scores were computed for each fold, and the mean of these scores served as the ultimate accuracy metric for the model. Each model underwent training on a randomized split, utilizing 66% for training data and 33% for test data, with a fixed random state of one. The split was conducted at the image level, through the randomization of the samples amongst donors. Precision, recall, and F1-score were documented, alongside the accuracy from Repeated Stratified K-Fold validation. This classification process was repeated for each set of feature subsets determined by the previously mentioned feature refinement methods, inclusive of a final aggregation of all selected features. All supervised ML models, dimensionality reduction, cross-validation and performance scoring were implemented using Python3 SciKit Learn ML packages.

## 3 Experimental results

**FIGURE 2:**
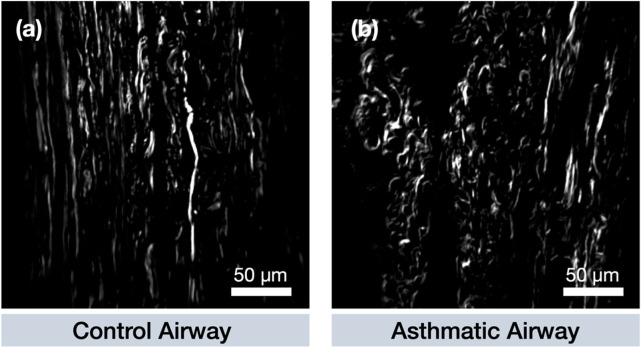
Representative SHG images of fibrillar collagen deposition in airways from **(a)** control and **(a)** asthmatic donors. A more uniform collagen fibre arrangement is evident in control airways, while a more fragmented structure is typically present in asthmatic cases. **(a)**. Performance using all 80 extracted features.

Representative SHG images depicting the collagen organization in non-asthmatic control and an asthmatic airway are depicted in Figure 2.

### 3.1 Performance using all 80 extracted features

Though it is not advisable to supply simple supervised classification models with many more features than positive (asthmatic) cases available for classification, our initial objective was to investigate the functionality of using all 80 extracted gray-level textures. An overall k-fold cross-validation accuracy of 88% ± 1% in the classification of asthmatic airways vs. control airways was observed with a macro-averaged ROC-AUC of 0.98 ± 0.0, for k = 10, as summarized in Supplementary Figure S1. The model exhibited a higher precision of 0.88 ± 0.0 reflected in the classification of asthmatic airways in comparison to control airways.

### 3.2 Performance using refined Feature sets

Four feature refinement methods were tested and four SVM models were independently trained on the feature subsets identified by each refinement method. The selected features of each refinement method are shown in Table 1.

**TABLE 1:** Textural features included after the application of each feature refinement method.

Matrix		GLCM	NGTDM	GLSZM	GLRLM	GLDLM
ANOVA F Test - Filter Method (10 features)						
Feature		• Correlation • Maximal Correlation Coefficient	• Busyness	• Large Area Emphasis • Large Area Low Gray Level Zone Emphasis • Size Zone Variance	• Short Run Low Gray Emphasis • Gray Level Non-Uniformity Normalized • Gray Level Non-Uniformity • Run Entropy	• (no selected features)
Permutation Importance (PI) – Embedded Method (9 features)						
Feature		• Joint Entropy • Difference Entropy • Inverse Difference Normalized	• (no features selected)	• Small Area Emphasis • Size Zone Non-Uniformity Normalized	• Run Variance • Long Run Low Gray Emphasis • High Gray Level Run Emphasis	• Low Gray Level Emphasis
Recursive Feature Elimination (RFE) - Wrapper Method (7 features)						
Feature		• Correlation	• (no features selected)	• Large Area Emphasis • Large Area Low Gray Level Zone Emphasis • Size Zone Percentage	• Short Run Low Gray Emphasis • Gray Level Non-Uniformity Normalized • Run Entropy	• (no features selected)
Coefficient of Variation Method (CoV) - (17 features selected)						
Feature		• Autocorrelation • Cluster Prominence • Cluster Shade • Cluster Tendency	• Coarseness • Busyness	• Large Area Emphasis • High Gray Level Zone Emphasis • Small Area High Gray Level Zone Emphasis • Gray Level Variance • Size Zone Variance	• Short Run High Gray Emphasis • Gray Level Variance • Run Variance	• High Gray Level Emphasis • Small Dependence High Gray Level Emphasis • Gray Level Variance

GLCM: Gray-level co-occurrence matrix. GLSZM: Gray Level Size Zone Matrix. GLRLM: Gray Level Run Length Matrix. GLDM: Gray Level Dependence Matrix. NGTDM: neighbouring gray tone difference matrix.

**FIGURE 3:**
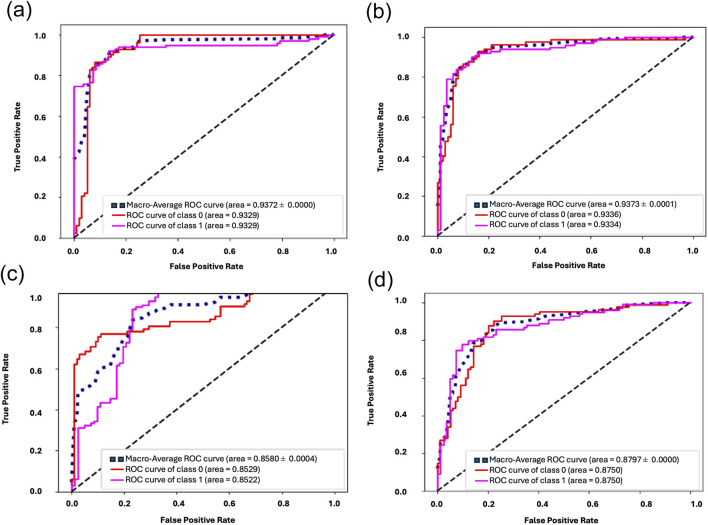
**(a–d)** Performance metrics for each subset of refined features**.** ROC-AUC obtained for each feature subset: **(a)** Filter Method–ANOVA F Test, **(b)** Wrapper Method–Recursive Feature Elimination, **(c)** Embedded Method–Permutation Importance and **(d)** Coefficient of Variation Method.

The performance achieved by each trained model on the four feature subsets are shown in Figure 3 and Table 2. Refined features selected by ANOVA F-Test (Filter Method) and Recursive Feature Elimination (Wrapper method) demonstrated high predictive importance, presenting optimal classification metrics of 86% ± 1% and 87% ± 2% accuracies respectively. With a selection of only seven features in total, RFE was capable of delineating between remodeled and control airways. SVM trained on RFE features boasted a k-Fold Cross Validation accuracy of 87% ± 2% with a Macro-Averaged ROC-AUC of 0.94 ± 0.0. The ANOVA F-test achieved a similar performance, with k-Fold Cross Validation accuracy of 86% ± 1% and Macro-Averaged ROC-AUC of 0.94 ± 0.0. In comparison, models trained on features selected by PI and CV achieved lower accuracies, with respective K-Fold Cross Validation accuracies of 80% ± 1% and 82% ± 1% and ROC-AUCs of 0.86 ± 0.0 and 0.88 ± 0.0. Confusion matrices for each classifier are found in Supplementary Figure S2.

**TABLE 2:** Summary of performance metrics of SVM trained on each of the four distinct feature subsets.

Method	Status	Precision	Recall	F1-score	Best Accuracy
Filter - ANOVA F	Control	0.84	0.83	0.84	86%
	Asthmatic	0.88	0.88	0.88	
Wrapper – RFE	Control	0.84	0.83	0.84	87%
	Asthmatic	0.88	0.88	0.88	
Embedded – PI	Control	0.81	0.81	0.81	80%
	Asthmatic	0.86	0.86	0.86	
CV	Control	0.80	0.79	0.80	82%
	Asthmatic	0.85	0.86	0.85	

RFE: Recursive Feature Elimination. PI: Permutation Importance. CV: coefficient of variance.

### 3.3 Performance using Pooled set of 33 features

**FIGURE 4:**
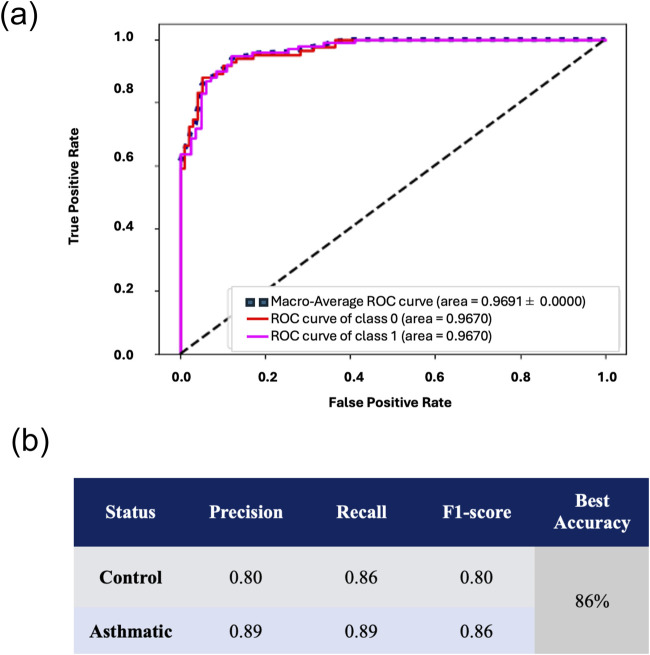
Performance metrics for 33 Pooled Features. Performance metrics of a trained SVM using all 33 Pooled features, showcasing **(a)** observed macro-averaged ROC-AUC of 0.9691 ± 0.00 and **(b)** precision, recall, and f1-scores for both asthmatic and control classes.

To improve classification accuracy and mitigate the risk of overfitting, the final subset of tested features comprised a pooling of all features identified using the four feature refinement methods. Redundant features common to all methods were eliminated, establishing a subset of 33 unique gray-level textural features (listed in Supplementary Tables S4, 5). High classification performance metrics of asthmatic and control collagen morphologies were observed, with a k-fold cross-validation accuracy of 86% ± 1% and Macro-Averaged ROC-AUC of 0.97 ± 0.0. The confusion matrix for pooled features is shown Supplementary Figure S3. Concerning the F1 scores in Figure 4, the model exhibited an F1-score of 0.83 for NA (control) indicating the model’s difficulty in accurately predicting the positive instances of NA (control).

## 4 Discussion

Insight into the structural alterations in ECM is essential to understanding disease pathology. In this study, we demonstrate the utility of supervised machine learning coupled with texture-based analysis in elucidating ECM remodeling in asthma. Specifically, we highlight that the deposition and morphology of fibrillar collagen in control and remodeled airways vastly differ. We apply first- and second-order statistical texture features to quantitatively assess the variance in collagen morphology between non-asthmatic control and asthmatic or remodeled airway states. By identifying distinct traits specific to control and remodeled collagen, we can objectively track ECM modifications in a diseased environment, specifically in asthma. Given that the interactions between the microenvironment and its cellular counterparts are slowly becoming understood as a driving force between tissue and chronic disease, there is a need to establish quantitative methodologies capable of tracking said morphological changes.

Gray-level texture analysis presents an effective approach to monitoring tissue ECM alterations, especially in the structure and morphology of the collagen network. In a broader context, the five textural matrices have been commonly applied independently to track textural features denoting changes in disease states of varying imaging modalities such as MRI, CT images, ultrasound, x-ray microscopic cellular and histopathological images (Alvarenga et al., 2010; Chabat et al., 2003; Gibbs and Turnbull, 2003; Herlidou et al., 2004; Moura et al., 2022). Success in the evaluation of collagen deposition was demonstrated using SHG and GLCM in tracking the aging of tissues and the onset of pancreatic cancer (Hu et al., 2012). To provide an exhaustive overview of applicable features, features derived from five gray-level texture families (GLCM, GLRLM, GLDM, GLSZM, NGTDM) were evaluated. Though the operation of some of the matrices is deemed unconventional for tracking biological systems, the mathematical approach for feature characterization can easily be extrapolated to evaluate any convention, providing novelty to the analysis conducted. A combination of said textural matrices has been applied in examples of grain classification, dosiomics in radiation therapy and x-ray imaging (De Moura et al., 2020; Placidi et al., 2021; Robert Singh and Chaudhury, 2020).

The number of samples relative to the number of features is crucial. Using a subset of eighty features for a sample size of 549 is justifiable, however, we increase the dimensionality of the dataset at the cost of computational and classification complexity. Regardless of the SVM classifiers exhibiting optimal F1, precision, and recall scores for each class when supplied with all 80 textural features, a near-perfect macro-averaged ROC of 0.98 is indicative of poor generalization. Standard approaches to address overfit models involve tasks such as eliminating outliers from the dataset, expanding the dataset size, rectifying class imbalances, and/or enhancing feature refinement. Due to sample limitations, increasing the size of the dataset is not a feasible option for our study. Using data engineering methods, such as image mirroring and stretching to grow the dataset would negatively impact the collagen morphologies unique to both the remodeled asthmatic airways and control airways. With an evenly balanced dataset of 296 images of asthmatic remodeled airways and 253 of control airways, the threat of class imbalance can be further eliminated.

To better understand the analysis of collagen morphologies using gray-level textures, it is crucial to identify how the selected features capture biological changes such as collagen fragmentation, fiber alignment, and disease progression. As expected, all four FOS features were eliminated by every refinement method. FOS primarily represents pixel intensity distributions without spatial context and thus fails to capture the broader structural patterns essential for distinguishing healthy from asthmatic tissues.

ANOVA F-Test and RFE (our two best-performing subsets) prioritized GLCM, GLSZM, GLRLM, and NGTDM features because these metrics reflect organization and continuity (or fragmentation) of fibrillar collagen bundles. For instance, GLCM correlation measures the likelihood of repetitive patterns, which is lower in asthmatic airways where collagen fibers become disorganized and fragmented. GLSZM features (e.g., large area emphasis) relate to the size and clustering of fibrotic collagen bundles—often larger, more irregular, and fragmented in diseased states. Similarly, GLRLM features capture consecutive pixel runs along specific directions; higher short-run emphasis, for example, signifies short segments of collagen indicative of pathological breakdown.

In line with these findings, other studies using large radiomics datasets (Moura et al., 2022; Zhang et al., 2023) confirm the necessity of feature refinement when dealing with high-dimensional feature spaces. While LASSO regression is common in radiomics, we compared four different refinement methods (ANOVA, RFE, PI, and CoV) to ensure an unbiased understanding of feature relevance in differentiating remodeling patterns in asthma. RFE’s reliance on supervised models can risk overfitting, but the final chosen features still yielded robust classification performance. Conversely, filter methods like ANOVA directly measure the separation between classes, selecting features that reflect morphological distinctions (e.g., fibril alignment vs. disorganized deposition) without depending on a predictive model.

Our key objective is to explain the observed collagen remodeling rather than merely optimize classification metrics. Collagen I and III, which provide tensile strength and regulate cellular responses, often exhibit fragmented or over-deposited patterns in asthmatic airways (Burgstaller et al., 2017). Pathologic changes in collagen fibril formation can negatively impair cell polarity and alignment (Rozario and DeSimone, 2010). These changes are strongly associated with the pathogenesis and progression of airway disease (Liu Y. et al., 2021). Hence, features capturing high entropy, large fragmented zones, or reduced fiber orientation (e.g., run entropy, size zone variance, and busyness) are biologically meaningful indicators of disease progression. We found that GLSZM and GLRLM metrics were particularly sensitive to these changes, highlighting the thicker, more variably oriented bundles commonly observed in remodeled airways.

Though deep-learning methods and transfer learning have proven to be successful at denoting textural variation and outperforming classical models in accuracy, they often obscure which specific image features drive predictions. With them aim of improving classification accuracy, Davydko et al., propose the aggregation of all five textural matrices using a Feature-Constructor neural network structure comprised of an encoder, encoded features aggregator and neural networks (Davydko et al., 2021). The hybrid classifiers based on CNN, LSOF, GMDH are applied to characterize pneumonic lesion types of COVID-19. Though a total accuracy of 0.96 was achieved, the optimal aggregation of the five textural matrices remains blinded to the user, shielding the contribution of each specified feature. The authors state that though the application of all five matrices are powerful textural descriptors, they do not all necessarily apply in the context of their classification problem. Hence, insight into the exact feature subsets leveraged for high classification accuracy is necessitated (Davydko et al., 2021). In contrast, our approach uses hand-selected gray-level features to quantitatively link machine-learning outputs to actual collagen changes in the tissue microenvironment. This transparency helps validate the findings from a biological standpoint, providing a clearer picture of how collagen remodeling manifests in the ECM and influences disease severity.

To conclude, feature refinement was imperative to reduce our initial set of 80 features to a manageable subset that best reflects collagen’s structural and morphological alterations. GLSZM and GLRLM in particular provided strong discriminative power, offering insight into bundle size, fragmentation, and orientation. While each texture matrix has limitations—such as GLSZM’s lack of orientation or GLCM’s reliance on only four directional angles—combining multiple matrices yields a robust, multifaceted assessment of fibril arrangement. Ultimately, this approach enables a clearer biological interpretation of collagen remodeling in asthma and underscores the necessity of selecting textural features that specifically capture pathological fiber changes.

Despite the novelty of employing a substantial number of gray texture features, incorporating local binary pattern textures or Law’s textures could aid in addressing pertinent information gaps. Furthermore, the features computed in this study lacked scale invariance. Hence, alterations in image magnification, variations in ROI and observation window sizes, pose a threat to the calculated features. To ensure consistency, a set of images should be captured at the same magnification, as the impact of differing ROIs or observation window sizes was evident. The deliberate selection of ROIs, through either manual intervention or automated methods, could curb this impact.

All presented models (trained using all 80 features, subsets of refined features and pooled features) encountered challenges when classifying the collagen morphology of control airways. In the applied dataset, significant overlaps in the architecture of collagen in both remodeled asthmatic and control airways were evident. Even with the supply of high predictive features, the model presented challenges to separate amongst disease groups. This demonstrates the difficulty in the exploration of the cellular microenvironment when samples are not from tightly controlled sources. Biological samples display a wider range of differences in collagen network morphology within groups. The probability of control collagen being misclassified as asthmatic airways was more prevalent than the reverse case. Though it is outside of the scope of this study, it would be pertinent to explore the relation of collagen distribution amongst individuals of varying sexes, ages and differing comorbidities. The lack of standardization of the collagen morphologies amongst our control donors could lead to added complexity in the classification task. Ideally, individualistic traits such as age and sex should be accounted for as they are potential factors driving differences in the collagen microstructure. However, the current work was limited in its sample size, curbing the exploration of the aforementioned. With an expanded dataset, these characteristics will be considered in future analysis. To better support the generalization of our proposed pipeline, the employed set of textural features should be tested on an independent secondary dataset of relevant SHG images. Though it is not presented in this work, the proposed pipeline has been applied to characterize collagen-characteristics in varying SHG datasets, analyzing the role of collagen morphology and structure in age-related changes of tendons, burn effects in skin, development of atherosclerosis and infarcted hearts.

The work presented facilitates the extrapolation in assessing the effectiveness of drug treatments in reversing the fibrotic response observed during the onset of asthma. With this proof of concept, clinicians could investigate the impact of these treatments on ECM remodeling and ascertain their efficacy in redirecting collagen accumulation in presuming a non-fibrotic environment.

## 5 Conclusion

Extracting textural features associated with collagen remodeling in biomedical tissues offers a more comprehensive and objective analysis of fibrotic responses, especially those related to changes in airway structures. Such metrics can be used to determine the extent of airway remodeling in obstructive lung diseases such as asthma or chronic obstructive pulmonary disease and/or the effects of potential novel therapies. The combination of label-free microscopy imaging and supervised machine learning approaches allowed us to demonstrate for the first time that gray-level-based textural variation of the fibrillar collagen can be a promising approach to identify subtle structural changes in asthmatics compared to non-asthmatics airways.

## Data Availability

The datasets presented in this article are not readily available due to the novelty of the SHG-imaged lung collagen dataset and its use for supporting research projects, the raw data will not be shared as of yet. Requests to access the datasets should be directed to Dr. Leila B. Mostaço-Guidolin, leila@sce.carleton.ca.
