# Supplementary material for: Classification of collagen remodeling in asthma using second-harmonic generation imaging, supervised machine learning and texture-based analysis
Source: Front Bioinform. 2025 Apr 17;5:1539936. doi: 10.3389/fbinf.2025.1539936 (PMC12043662; doi:10.3389/fbinf.2025.1539936)
Supplement: Supplementary file 1 [file DataSheet1.docx]

**TABLES**

**Supplemental Table 1.** Grey-Level Texture Feature Matrices, their description and features extracted.

| **Gray-level Texture Group** | **Overview of Operation** | **Computed Features** | |
| --- | --- | --- | --- |
| **GLCM** | The Gray-level Co-occurrence Matrix (GLCM) was originally introduced to capture the distribution of pixel intensities within a designated plane. The GLCM's computed values are influenced by the arrangement of structures within an image and counts the frequency of recurring pixel intensities.  In the GLCM, comparisons are drawn between a central pixel and its neighboring pixels at specific offsets within the matrix's defined direction. This results in a square matrix that quantifies the total occurrences of co-occurring pixel values for the specified direction, considering both forward and reverse orientations. To obtain a comprehensive representation of the texture content, four directional matrices are computed, summed, and averaged, all independent of the sample's orientation. The calculated matrix is subsequently used to compute texture features, with a total of 24 extracted features. The computed textural features serve as measures to track the overall pixel organization within an image by quantifying the relationships between pairs of pixel intensities. | -    Autocorrelation  -    Cluster Prominence  -    Cluster Shade  -    Cluster Tendency  -    Contrast  -    Correlation  -    Difference Entropy  -    Difference Variance  -    Dissimilarity  -    Energy  -    Entropy  -    Homogeneity 1  -    Homogeneity 2 | -    Inverse Difference Normalized  -    Maximum Probability  -    Sum Average  -    Sum Entropy  -    Sum Variance  -    Sum of Squares  -    Maximum Correlation Coefficient  -    Information Measure of Correlation 1  -    Information Measure of Correlation 2  -    Inverse Difference Moment Normalized |
| **GLSZM** | The Gray-Level Size Zone Matrix (GLSZM) was designed to tally the occurrences of various sizes of zones corresponding to each gray level within an image or region. Originally developed for characterizing cell texture, the GLSZM proves highly adept at quantifying image homogeneity, discerning speckle-like structures, and evaluating the structural composition of an image, including aspects such as the prevalence of small versus large regions.  Unique to the GLSZM, a zone is defined as a group of interconnected pixels sharing identical intensity values. It is a non-directional matrix that does not necessitate multiple passes over an image, nor the averaging of computed features to generate texture descriptors for the entire image. GLSZM computes a set of 16 features, relying on the size of the pixel zones as the basis of capturing the size of structures. | -    Small Area Emphasis  -    Large Area Emphasis  -    Low Gray Level Area Emphasis  -    High Gray Level Area Emphasis  -    Small Low Gray Area Emphasis  -    Small High Gray Area Emphasis  -    Large Low Gray Area Emphasis  -    Large High Gray Area Emphasis  -    Gray Level Variance | -    Gray Level Non-Uniformity  -    Gray Level Non-Uniformity Normalized  -    Size Zone Variance  -    Size Zone Non-Uniformity  -    Size Zone Non-Uniformity Normalized  -    Size Zone Percentage  -    Size Zone Entropy |
| **GLRLM** | Gray-Level Run Length Matrix (GLRLM) comprises a set of texture features that focus on analyzing the run lengths of gray levels within an image. GLRLM is particularly well-suited for assessing linear structures, as it quantifies the length of consecutive runs of pixel intensities.   GLRLM operates by counting the number of sequentially connected pixels in a specified direction, where connected pixels share the same gray intensity, forming what is known as a "run." Similar to GLCM, GLRLM is directional and can be constructed along four primary directions: horizontally (0 degrees), diagonally right (45 degrees), vertically (90 degrees), and diagonally left (135 degrees). Features are subsequently derived from each directional matrix and then averaged to generate texture descriptors for the entire image. A total of 16 features are computed. | -    Long High Gray Run Emphasis  -    Gray Level Variance  -    Gray Level Non-Uniformity  -    Gray Level Non-Uniformity Normalized  -    Run Length Variance  -    Run Length Non-Uniformity  -    Run Length Non-Uniformity Normalized  -    Run Percentage  -    Run Entropy | -    Short Run Emphasis  -    Long Run Emphasis  -    Low Gray Level Run Emphasis  -    High Gray Level Run Emphasis  -    Short Low Gray Run Emphasis  -    Short High Gray Run Emphasis  -    Long Low Gray Run Emphasis |
| **GLDM** | Gray-Level Dependence Matrix (GLDM) primarily evaluates the overall coarseness and/or fineness of an image.    Matrix construction involves tallying the number of dependent pixels within a defined neighborhood around each pixel in a region. A total of 15 features can be derived from GLDM, providing a means to track the coarseness or fineness of structures within an image by quantifying the likelihood of encountering pixels of the same intensity in proximity to one another. | -    Small Number Emphasis  -    Large Number Emphasis  -    Low Gray Level Count Emphasis  -    High Gray Level Count Emphasis  -    Small Number Low Gray Level Emphasis  -    Gray Level Non-Uniformity Normalized  -    Dependence Variance | -    Small Number High Gray Level Emphasis  -    Large Number Low Gray Level Emphasis  -    Large Number High Gray Level Emphasis  -    Gray Level Variance  -    Gray Level Non-Uniformity  -    Dependence Non-Uniformity  -    Dependence Non-Uniformity Normalized  -    Dependence Entropy |
| **NGTDM** | The Neighboring Gray Tone Difference Matrix (NGTDM) constitutes a set of texture features centered around assessing the disparity between the intensity of a pixel and the average intensity of its surrounding pixels. NGTDM originated from mimicking human perception of texture, taking inspiration from characteristics like coarseness, contrast, complexity, busyness, and strength. Its primary focus lies in gauging how prominently a texture stands out from its background and how perceivable the overall texture would be to a human observer. NGTDM does not concentrate on specific structural aspects, such as linear runs or regions, but instead, it excels in determining several fundamental texture attributes within an image. NGTDM examines the neighborhood surrounding a central pixel, calculating five distinct texture descriptors: coarseness, contrast, busyness, complexity, and strength. | -    Coarseness  -    Contrast  -      Busyness  -      Complexity  -      Strength |  |

GLCM: Gray-level co-occurrence matrix. GLSZM: Gray Level Size Zone Matrix. GLRLM: Gray Level Run Length Matrix. GLDM: Gray Level Dependence Matrix. NGTDM: Neighbouring Gray Tone Difference Matrix.

**Supplemental Table 2.** Feature statistics for all 80 features – Control Airways

| Gray-level Texture Feature | Mean | Std. Deviation | Minimum | Maximum |
| --- | --- | --- | --- | --- |
| Mean | 1.41 | 0.2 | 0.94 | 1.93 |
| Standard Deviation | 6.67 | 0.68 | 5.05 | 8.59 |
| Kurtosis | 201 | 42.75 | 128.6 | 365.9 |
| Skewness | 11.25 | 1 | 9.14 | 14.39 |
| GLCM - Autocorrelation | 43.16 | 9.01 | 23.55 | 71.82 |
| GLCM - Cluster Prominence | 5362468 | 1596124 | 2460261 | 1.04E+07 |
| GLCM - Cluster Shade | 23223 | 5922 | 11434 | 42902 |
| GLCM - Cluster Tendency | 163.5 | 34.69 | 93.41 | 268.5 |
| GLCM - Contrast | 14.42 | 2.68 | 8.38 | 22.07 |
| GLCM - Correlation | 0.85 | 0.02 | 0.77 | 0.89 |
| GLCM - Joint Average | 2.41 | 0.2 | 1.94 | 2.93 |
| GLCM - Joint Energy | 0.66 | 0.03 | 0.6 | 0.74 |
| GLCM - Joint Entropy | 2.09 | 0.18 | 1.62 | 2.57 |
| GLCM - Difference Average | 0.82 | 0.11 | 0.57 | 1.16 |
| GLCM - Difference Entropy | 1.2 | 0.09 | 0.94 | 1.43 |
| GLCM - Difference Variance | 13.8 | 2.46 | 8.03 | 20.52 |
| GLCM - Inverse Difference Moment | 0.87 | 0.01 | 0.84 | 0.91 |
| GLCM - Inverse Difference Moment Normalized | 1 | 3.91E-05 | 1 | 1 |
| GLCM - Inverse Difference | 0.89 | 0.01 | 0.86 | 0.92 |
| GLCM - Inverse Difference Normalized | 1 | 4.02E-04 | 1 | 1 |
| GLCM - Inverse Variance | 0.07 | 0.01 | 0.05 | 0.08 |
| GLCM - Informational Measure of Correlation 1 | -0.34 | 0.01 | -0.38 | -0.3 |
| GLCM - Informational Measure of Correlation 2 | 0.74 | 0.02 | 0.69 | 0.79 |
| GLCM - Maximal Correlation Coefficient | 0.87 | 0.02 | 0.83 | 0.91 |
| GLCM - Maximum Probability | 0.81 | 0.02 | 0.77 | 0.86 |
| GLCM - Sum Average | 4.82 | 0.39 | 3.89 | 5.87 |
| GLCM - Sum Entropy | 1.61 | 0.13 | 1.27 | 1.94 |
| GLCM - Sum Of Squares | 44.95 | 9.32 | 25.56 | 73.96 |
| GLSZM - Small Area Emphasis | 0.98 | 2.58E-03 | 0.97 | 0.98 |
| GLSZM - Large Area Emphasis | 1.5 | 0.16 | 1.39 | 2.2 |
| GLSZM - Low Gray Level Zone Emphasis | 0.91 | 0.01 | 0.88 | 0.93 |
| GLSZM - High Gray Level Zone Emphasis | 49.34 | 10.07 | 27.93 | 81.57 |
| GLSZM - Small Area Low Gray Level Zone Emphasis | 0.91 | 0.01 | 0.88 | 0.93 |
| GLSZM - Small Area High Gray Level Zone Emphasis | 46.15 | 9.53 | 26.32 | 77.26 |
| GLSZM - Large Area Low Gray Level Zone Emphasis | 1.03 | 0.04 | 0.97 | 1.15 |
| GLSZM - Large Area High Gray Level Zone Emphasis | 72.11 | 13.44 | 41.15 | 114.6 |
| GLSZM - Gray Level Variance | 44.07 | 9.32 | 24.92 | 74.02 |
| GLSZM - Gray Level Non-Uniformity | 200789 | 5835 | 187171 | 215418 |
| GLSZM - Gray Level Non-Uniformity Normalized | 0.82 | 0.02 | 0.76 | 0.87 |
| GLSZM - Size Zone Variance | 0.38 | 0.15 | 0.28 | 1.05 |
| GLSZM - Size Zone Non-Uniformity | 233393 | 3245 | 226404 | 242045 |
| GLSZM - Size Zone Non-Uniformity Normalized | 0.95 | 0.01 | 0.93 | 0.96 |
| GLSZM - Size Zone Percentage | 0.94 | 0.01 | 0.93 | 0.96 |
| GLSZM - Size Zone Entropy | 1.06 | 0.11 | 0.8 | 1.35 |
| GLRLM - Short Run Emphasis | 0.76 | 0.01 | 0.73 | 0.79 |
| GLRLM - Long Run Emphasis | 655.6 | 166.7 | 349.2 | 1284 |
| GLRLM - Low Gray Level Run Emphasis | 0.28 | 0.01 | 0.25 | 0.31 |
| GLRLM - High Gray Level Run Emphasis | 306.6 | 58.83 | 197.3 | 489.1 |
| GLRLM - Short Run Low Gray Emphasis | 0.09 | 3.87E-03 | 0.09 | 0.11 |
| GLRLM - Short Run High Gray Emphasis | 300.5 | 57.2 | 194.4 | 477 |
| GLRLM - Long Run Low Gray Emphasis | 653.9 | 166.8 | 347.9 | 1283 |
| GLRLM - Long Run High Gray Emphasis | 1064 | 189.4 | 672.9 | 1628 |
| GLRLM - Gray Level Non-Uniformity | 3898 | 401.1 | 3299 | 5238 |
| GLRLM - Gray Level Non-Uniformity Normalized | 0.09 | 0.01 | 0.08 | 0.12 |
| GLRLM - Run Length Non-Uniformity | 23420 | 2694 | 17775 | 30621 |
| GLRLM - Run Length Non-Uniformity Normalized | 0.55 | 0.02 | 0.5 | 0.59 |
| GLRLM - Run Length Percentage | 0.17 | 0.02 | 0.13 | 0.21 |
| GLRLM - Gray Level Variance | 223.9 | 41.29 | 145.7 | 354.9 |
| GLRLM - Run Variance | 614.4 | 160.4 | 329.1 | 1200 |
| GLRLM - Run Entropy | 5.89 | 0.09 | 5.67 | 6.14 |
| GLDM - Small Dependence Emphasis | 0.09 | 0.01 | 0.07 | 0.12 |
| GLDM - Large Dependence Emphasis | 65.41 | 1.45 | 61.81 | 69.17 |
| GLDM - Low Gray Level Emphasis | 0.86 | 0.01 | 0.83 | 0.9 |
| GLDM - High Gray Level Emphasis | 50.95 | 10.26 | 29.06 | 83.27 |
| GLDM - Small Dependence Low Gray Level Emphasis | 0.02 | 4.73E-04 | 0.02 | 0.02 |
| GLDM - Small Dependence High Gray Level Emphasis | 43.54 | 9.19 | 24.69 | 73.25 |
| GLDM - Large Dependence Low Gray Level Emphasis | 64.42 | 1.53 | 60.8 | 68.68 |
| GLDM - Large Dependence High Gray Level Emphasis | 150.8 | 15.2 | 118.6 | 190.8 |
| GLDM - Gray Level Variance | 44.9 | 9.31 | 25.5 | 73.76 |
| GLDM - Gray Level Non-Uniformity | 188392 | 6907 | 172691 | 206621 |
| GLDM - Gray Level Non-Uniformity Normalized | 0.72 | 0.03 | 0.66 | 0.79 |
| GLDM - Dependence Variance | 6.65 | 0.52 | 5.33 | 7.93 |
| GLDM - Dependence Non-Uniformity | 144692 | 8353 | 124618 | 168788 |
| GLDM - Dependence Non-Uniformity Normalized | 0.55 | 0.03 | 0.48 | 0.64 |
| GLDM - Dependence Entropy | 2.3 | 0.17 | 1.82 | 2.68 |
| NGTDM - Coarseness | 1.36E-03 | 2.86E-04 | 8.94E-04 | 2.48E-03 |
| NGTDM - Contrast | 9.30E-04 | 2.08E-04 | 4.85E-04 | 1.56E-03 |
| NGTDM - Busyness | 1.71 | 0.36 | 1.4 | 3.15 |
| NGTDM - Complexity | 71588 | 11171 | 43380 | 97411 |
| NGTDM - Strength | 5.31 | 0.73 | 3.81 | 7.43 |

GLCM: Gray-level co-occurrence matrix. GLSZM: Gray Level Size Zone Matrix. GLRLM: Gray Level Run Length Matrix. GLDM: Gray Level Dependence Matrix. NGTDM: Neighbouring Gray Tone Difference Matrix.

**Supplemental Table 3.** Feature statistics for all 80 features – Asthmatic Airways

| Gray-Level Texture Feature | Mean | Std. Deviation | Minimum | Maximum |
| --- | --- | --- | --- | --- |
| Mean | 1.5 | 0.23 | 0.94 | 2 |
| Standard Deviation | 7.2 | 0.72 | 5.3 | 8.9 |
| Kurtosis | 187 | 43 | 120 | 340 |
| Skewness | 11 | 1.2 | 9 | 15 |
| GLCM - Autocorrelation | 50 | 10 | 27 | 79 |
| GLCM - Cluster Prominence | 6677861 | 1792736 | 3146280 | 1.26E+07 |
| GLCM - Cluster Shade | 28655 | 6769 | 14965 | 48608 |
| GLCM - Cluster Tendency | 193 | 39 | 102 | 299 |
| GLCM - Contrast | 14 | 2.3 | 8.1 | 20 |
| GLCM - Correlation | 0.82 | 0.023 | 0.8 | 0.91 |
| GLCM - Joint Average | 2.5 | 0.23 | 1.9 | 3.1 |
| GLCM - Joint Energy | 0.68 | 0.031 | 0.59 | 0.76 |
| GLCM - Joint Entropy | 2 | 0.21 | 1.5 | 2.6 |
| GLCM - Difference Average | 0.8 | 0.11 | 0.54 | 1.1 |
| GLCM - Difference Entropy | 1.2 | 0.11 | 0.88 | 1.4 |
| GLCM - Difference Variance | 13 | 2.2 | 7.9 | 18 |
| GLCM - Inverse Difference Moment | 0.88 | 0.014 | 0.84 | 0.91 |
| GLCM - Inverse Difference Moment Normalized | 1 | 0.000033 | 1 | 1 |
| GLCM - Inverse Difference | 0.89 | 0.013 | 0.86 | 0.92 |
| GLCM - Inverse Difference Normalized | 1 | 0.00039 | 1 | 1 |
| GLCM - Inverse Variance | 0.063 | 0.0065 | 0.044 | 0.079 |
| GLCM - Informational Measure of Correlation 1 | -0.35 | 0.014 | -0.39 | -0.32 |
| GLCM - Informational Measure of Correlation 2 | 0.75 | 0.024 | 0.68 | 0.8 |
| GLCM - Maximal Correlation Coefficient | 0.89 | 0.016 | 0.85 | 0.92 |
| GLCM - Maximum Probability | 0.82 | 0.019 | 0.77 | 0.87 |
| GLCM - Sum Average | 4.9 | 0.46 | 3.9 | 6.1 |
| GLCM - Sum Entropy | 1.6 | 0.15 | 1.2 | 2 |
| GLCM - Sum Of Squares | 52 | 10 | 28 | 79 |
| GLSZM - Small Area Emphasis | 0.98 | 0.0027 | 0.97 | 0.99 |
| GLSZM - Large Area Emphasis | 1.7 | 0.16 | 1.2 | 2.2 |
| GLSZM - Low Gray Level Zone Emphasis | 0.91 | 0.012 | 0.88 | 0.94 |
| GLSZM - High Gray Level Zone Emphasis | 55 | 11 | 30 | 85 |
| GLSZM - Small Area Low Gray Level Zone Emphasis | 0.91 | 0.012 | 0.88 | 0.94 |
| GLSZM - Small Area High Gray Level Zone Emphasis | 52 | 10 | 28 | 81 |
| GLSZM - Large Area Low Gray Level Zone Emphasis | 1 | 0.029 | 0.95 | 1.1 |
| GLSZM - Large Area High Gray Level Zone Emphasis | 78 | 15 | 46 | 120 |
| GLSZM - Gray Level Variance | 51 | 10 | 28 | 77 |
| GLSZM - Gray Level Non-Uniformity | 203188 | 6718 | 185238 | 219353 |
| GLSZM - Gray Level Non-Uniformity Normalized | 0.82 | 0.023 | 0.76 | 0.88 |
| GLSZM - Size Zone Variance | 0.54 | 0.14 | 0.15 | 0.97 |
| GLSZM - Size Zone Non-Uniformity | 235481 | 3349 | 227925 | 244103 |
| GLSZM - Size Zone Non-Uniformity Normalized | 0.95 | 0.0063 | 0.93 | 0.97 |
| GLSZM - Size Zone Percentage | 0.95 | 0.0071 | 0.93 | 0.97 |
| GLSZM - Size Zone Entropy | 1 | 0.12 | 0.73 | 1.4 |
| GLRLM - Short Run Emphasis | 0.77 | 0.011 | 0.74 | 0.79 |
| GLRLM - Long Run Emphasis | 731 | 194 | 383 | 1315 |
| GLRLM - Low Gray Level Run Emphasis | 0.27 | 0.012 | 0.24 | 0.3 |
| GLRLM - High Gray Level Run Emphasis | 359 | 63 | 232 | 553 |
| GLRLM - Short Run Low Gray Emphasis | 0.097 | 0.0045 | 0.082 | 0.1 |
| GLRLM - Short Run High Gray Emphasis | 350 | 62 | 227 | 543 |
| GLRLM - Long Run Low Gray Emphasis | 730 | 194 | 381 | 1314 |
| GLRLM - Long Run High Gray Emphasis | 1172 | 196 | 793 | 1840 |
| GLRLM - Gray Level Non-Uniformity | 4244 | 381 | 2852 | 4777 |
| GLRLM - Gray Level Non-Uniformity Normalized | 0.099 | 0.0073 | 0.078 | 0.11 |
| GLRLM - Run Length Non-Uniformity | 23270 | 3024 | 15880 | 30799 |
| GLRLM - Run Length Non-Uniformity Normalized | 0.56 | 0.017 | 0.51 | 0.59 |
| GLRLM - Run Length Percentage | 0.16 | 0.017 | 0.12 | 0.21 |
| GLRLM - Gray Level Variance | 259 | 46 | 169 | 393 |
| GLRLM - Run Variance | 689 | 185 | 355 | 1251 |
| GLRLM - Run Entropy | 6 | 0.084 | 5.8 | 6.2 |
| GLDM - Small Dependence Emphasis | 0.092 | 0.01 | 0.066 | 0.12 |
| GLDM - Large Dependence Emphasis | 66 | 1.5 | 62 | 70 |
| GLDM - Low Gray Level Emphasis | 0.87 | 0.016 | 0.83 | 0.9 |
| GLDM - High Gray Level Emphasis | 58 | 11 | 32 | 89 |
| GLDM - Small Dependence Low Gray Level Emphasis | 0.017 | 0.00051 | 0.016 | 0.018 |
| GLDM - Small Dependence High Gray Level Emphasis | 50 | 10 | 27 | 76 |
| GLDM - Large Dependence Low Gray Level Emphasis | 65 | 1.6 | 61 | 70 |
| GLDM - Large Dependence High Gray Level Emphasis | 160 | 18 | 124 | 211 |
| GLDM - Gray Level Variance | 52 | 10 | 28 | 79 |
| GLDM - Gray Level Non-Uniformity | 191812 | 7505 | 172785 | 210423 |
| GLDM - Gray Level Non-Uniformity Normalized | 0.73 | 0.029 | 0.66 | 0.8 |
| GLDM - Dependence Variance | 6.5 | 0.6 | 5 | 7.9 |
| GLDM - Dependence Non-Uniformity | 148930 | 8473 | 127843 | 174500 |
| GLDM - Dependence Non-Uniformity Normalized | 0.57 | 0.032 | 0.49 | 0.67 |
| GLDM - Dependence Entropy | 2.2 | 0.19 | 1.7 | 2.7 |
| NGTDM - Coarseness | 0.0016 | 0.00033 | 0.001 | 0.0028 |
| NGTDM - Contrast | 0.00094 | 0.00021 | 0.00048 | 0.0015 |
| NGTDM - Busyness | 2.0 | 0.26 | 1.1 | 2.5 |
| NGTDM - Complexity | 71724 | 10268 | 49518 | 98354 |
| NGTDM - Strength | 5.6 | 0.8 | 3.8 | 7.6 |

GLCM: Gray-level co-occurrence matrix. GLSZM: Gray Level Size Zone Matrix. GLRLM: Gray Level Run Length Matrix. GLDM: Gray Level Dependence Matrix. NGTDM: Neighbouring Gray Tone Difference Matrix.

**Supplemental Table 4.** Feature statistics for Pooled 33 features – Control Airways

| Gray-level Texture Feature | Mean | Std. Deviation | Minimum | Maximum |
| --- | --- | --- | --- | --- |
| GLCM - Autocorrelation | 43.16 | 9.005 | 23.55 | 71.82 |
| GLCM - Cluster Prominence | 5362468 | 1596124 | 2460261 | 10385491 |
| GLCM - Cluster Shade | 23223 | 5922 | 11434 | 42902 |
| GLCM - Cluster Tendency | 163.5 | 34.69 | 93.41 | 268.5 |
| GLCM - Correlation | 0.8538 | 0.02435 | 0.7722 | 0.8892 |
| GLCM - Joint Entropy | 2.094 | 0.1815 | 1.618 | 2.567 |
| GLCM - Difference Entropy | 1.196 | 0.09462 | 0.9364 | 1.425 |
| GLCM - Inverse Difference Normalized | 0.997 | 0.0004015 | 0.9957 | 0.9979 |
| GLCM - Maximal Correlation Coefficient | 0.8714 | 0.01604 | 0.8334 | 0.9072 |
| GLSZM - Small Area Emphasis | 0.9781 | 0.002579 | 0.9722 | 0.9841 |
| GLSZM - Large Area Emphasis | 1.497 | 0.1585 | 1.391 | 2.197 |
| GLSZM - High Gray Level Zone Emphasis | 49.34 | 10.07 | 27.93 | 81.57 |
| GLSZM - Small Area High Gray Level Zone Emphasis | 46.15 | 9.534 | 26.32 | 77.26 |
| GLSZM - Large Area Low Gray Level Zone Emphasis | 1.026 | 0.03546 | 0.969 | 1.149 |
| GLSZM - Gray Level Variance | 44.07 | 9.316 | 24.92 | 74.02 |
| GLSZM - Size Zone Variance | 0.3779 | 0.1523 | 0.284 | 1.047 |
| GLSZM - Size Zone Non-Uniformity | 233393 | 3245 | 226404 | 242045 |
| GLSZM - Size Zone Percentage | 0.9417 | 0.006999 | 0.9252 | 0.959 |
| GLRLM - High Gray Level Run Emphasis | 306.6 | 58.83 | 197.3 | 489.1 |
| GLRLM - Short Run Low Gray Emphasis | 0.09206 | 0.003871 | 0.08765 | 0.1076 |
| GLRLM - Short Run High Gray Emphasis | 300.5 | 57.2 | 194.4 | 477 |
| GLRLM - Long Run Low Gray Emphasis | 653.9 | 166.8 | 347.9 | 1283 |
| GLRLM - Gray Level Non-Uniformity | 3898 | 401.1 | 3299 | 5238 |
| GLRLM - Gray Level Non-Uniformity Normalized | 0.09247 | 0.007508 | 0.08089 | 0.1199 |
| GLRLM - Gray Level Variance | 223.9 | 41.29 | 145.7 | 354.9 |
| GLRLM - Run Variance | 614.4 | 160.4 | 329.1 | 1200 |
| GLRLM - Run Entropy | 5.888 | 0.09489 | 5.674 | 6.144 |
| GLDM - Low Gray Level Emphasis | 0.8596 | 0.01439 | 0.8277 | 0.8964 |
| GLDM - High Gray Level Emphasis | 50.95 | 10.26 | 29.06 | 83.27 |
| GLDM - Small Dependence High Gray Level Emphasis | 43.54 | 9.187 | 24.69 | 73.25 |
| GLDM - Gray Level Variance | 44.9 | 9.305 | 25.5 | 73.76 |
| NGTDM - Coarseness | 0.001363 | 0.0002859 | 0.0008939 | 0.002478 |
| NGTDM - Busyness | 1.712 | 0.364 | 1.403 | 3.149 |

**Supplemental Table 5.** Feature statistics for Pooled 33 features – Asthmatic Airways

| Gray-Level Texture Feature | Mean | Std. Deviation | Minimum | Maximum |
| --- | --- | --- | --- | --- |
| GLCM - Autocorrelation | 50.14 | 10.42 | 27.13 | 79.15 |
| GLCM - Cluster Prominence | 6677861 | 1792736 | 3146280 | 12630874 |
| GLCM - Cluster Shade | 28655 | 6769 | 14965 | 48608 |
| GLCM - Cluster Tendency | 193 | 39.24 | 102.4 | 298.7 |
| GLCM - Correlation | 0.8249 | 0.0235 | 0.8001 | 0.9117 |
| GLCM - Joint Entropy | 2.026 | 0.2141 | 1.494 | 2.581 |
| GLCM - Difference Entropy | 1.157 | 0.1091 | 0.8845 | 1.427 |
| GLCM - Inverse Difference Normalized | 0.997 | 0.0003858 | 0.9961 | 0.998 |
| GLCM - Maximal Correlation Coefficient | 0.8853 | 0.01621 | 0.8493 | 0.9239 |
| GLSZM - Small Area Emphasis | 0.9794 | 0.002672 | 0.9725 | 0.9862 |
| GLSZM - Large Area Emphasis | 1.687 | 0.1573 | 1.233 | 2.156 |
| GLSZM - High Gray Level Zone Emphasis | 55.48 | 11.17 | 30.49 | 84.97 |
| GLSZM - Small Area High Gray Level Zone Emphasis | 52.28 | 10.41 | 28.45 | 80.55 |
| GLSZM - Large Area Low Gray Level Zone Emphasis | 0.9962 | 0.02915 | 0.9501 | 1.109 |
| GLSZM - Gray Level Variance | 50.51 | 10.18 | 27.53 | 77.44 |
| GLSZM - Size Zone Variance | 0.5394 | 0.1428 | 0.1512 | 0.97 |
| GLSZM - Size Zone Non-Uniformity | 235481 | 3349 | 227925 | 244103 |
| GLSZM - Size Zone Percentage | 0.9465 | 0.007082 | 0.9293 | 0.9665 |
| GLRLM - High Gray Level Run Emphasis | 358.7 | 62.58 | 231.7 | 553.1 |
| GLRLM - Short Run Low Gray Emphasis | 0. 09698 | 0.004487 | 0.0819 | 0.1036 |
| GLRLM - Short Run High Gray Emphasis | 350.5 | 61.76 | 226.5 | 542.7 |
| GLRLM - Long Run Low Gray Emphasis | 730.4 | 194.2 | 381.3 | 1314 |
| GLRLM - Gray Level Non-Uniformity | 4244 | 381.4 | 2852 | 4777 |
| GLRLM - Gray Level Non-Uniformity Normalized | 0.09882 | 0.007332 | 0.07766 | 0.114 |
| GLRLM - Gray Level Variance | 259 | 45.65 | 168.9 | 392.7 |
| GLRLM - Run Variance | 689.5 | 185.1 | 355.4 | 1251 |
| GLRLM - Run Entropy | 5.997 | 0.08428 | 5.796 | 6.203 |
| GLDM - Low Gray Level Emphasis | 0.8664 | 0.01593 | 0.8258 | 0.9039 |
| GLDM - High Gray Level Emphasis | 57.69 | 11.5 | 32.22 | 89.04 |
| GLDM - Small Dependence High Gray Level Emphasis | 50.09 | 10.01 | 27.26 | 76.44 |
| GLDM - Gray Level Variance | 52.1 | 10.32 | 28.31 | 78.9 |
| NGTDM - Coarseness | 0.001559 | 0.0003308 | 0.001014 | 0.002848 |
| NGTDM - Busyness | 2.048 | 0.2572 | 1.136 | 2.465 |

**FIGURES**

**(c)**

| **Status** | **Precision** | **Recall** | **F1-score** | **Best Accuracy** |
| --- | --- | --- | --- | --- |
| **Control** | 0.84 | 0.85 | 0.84 | 88% |
| **Asthmatic** | 0.88 | 0.87 | 0.87 |  |

**Supplemental Figure 1.** Classification metrics of All 80 Features. Performance metrics of a trained SVM using all 80 features, showcasing (a) observed macro-averaged ROC-AUC of 0.9849 ± 0.00, (b) the confusion matrix and (c) the computed precision, recall, and f1-scores for both asthmatic and control classes.

**Supplemental Figure 2.** Confusion matrices of SVM supplied with feature subsets from each feature refinement method. Performance metrics of the model trained independently on the four feature subsets extracted from (a) Filter Method – ANOVA F Test, (b) Wrapper Method – Recursive Feature Elimination, (c) Embedded Method – Permutation Importance and (d) Coefficient of Variation Method. A: asthmatic. NA: Non-asthmatic (control) airways.

**Supplemental Figure 3.** Confusion Matrix of SVM supplied with 33 Pooled Features. NA: non-asthmatic (control) airways. A: Asthmatic airways.
